# Screen time, smartphone addiction, and executive-motor function in Tunisian middle school students

**DOI:** 10.3389/fpsyg.2026.1694094

**Published:** 2026-05-07

**Authors:** Mohamed Yaakoubi, Ahmed Ghorbel, Hiba Abdelkafi, Mohammed Issa Alsaeed, Mustapha Bouchiba, Liwa Masmoudi, Noureddine Ben Said, Alexander Woll, Swantje Scharenberg, Mohamed Moncef Kammoun, Omar Trabelsi, Adnene Gharbi

**Affiliations:** 1 Research Unit, Physical Activity, Sport and Health, UR18JS01, National Observatory of Sport, Tunis, Tunisia; 2 The High Institute of Sport and Physical Education, University of Jendouba, Kef, Tunisia; 3 Department of Education Sciences, The High Institute of Applied Studies in Humanities of Mahdia, University of Monastir, Mahdia, Tunisia; 4 Department of Biomechanics & Motor Behavior, College of Sport Science & Physical Activity, King Saud University, Riyadh, Saudi Arabia; 5 Laboratoire Hypoxie & Poumon, UMR INSERM U1272, Université Sorbonne Paris Nord, Bobigny, France; 6 The High Institute of Sport and Physical Education, University of Sfax, Sfax, Tunisia; 7 Institute of Sports and Sports Science, Karlsruhe Institute of Technology, Karlsruhe, Germany

**Keywords:** adolescence, attention, executive function, fine motor skills, handwriting, smartphone usage, Tunisia

## Abstract

This study advances beyond general screen time metrics by investigating the specific associations between smartphone addiction and objectively measuring daily screen time and core neurocognitive and graphomotor functions in adolescents. In a cross-sectional study involving 270 Tunisian middle school students (mean age = 14.8 ± 0.5 years; 54% male and 46% female students) recruited from three public middle schools in the region of Sfax, Tunisia, during routine school health assessments (December 2023–March 2024), participants were classified as smartphone-addicted (*n* = 120) or non-addicted (*n* = 150) using a standardized scale, and their actual screen time was recorded from their devices. All participants completed assessments measuring sustained attention, selective attention, handwriting quality and speed, and abstract reasoning. The results showed that the addicted group demonstrated significantly poorer sustained attention (correct items: 177 ± 16 vs. 195 ± 19.4, *p* < 0.001), more errors in selective attention (interference errors: 3.0 ± 2.0 vs. 2.38 ± 1.80, *p* < 0.001), and reduced handwriting quality (errors: 13.0 ± 8.0 vs. 10.24 ± 7.09, *p* = 0.016). On the other hand, no differences emerged in abstract reasoning or handwriting speed. Regression analyses confirmed that both higher addiction severity and longer screen time were significant negative predictors, in separate models, of sustained attention (*R*^2^ = 0.27–0.34, *p* < 0.001), selective attention (*R*^2^ = 0.15–0.16, *p* < 0.001), and handwriting quality (*R*^2^ = 0.06–0.07, *p* < 0.001), but not of abstract reasoning. These findings demonstrate that smartphone addiction and excessive screen time are both associated with significant deficits in key attentional and motor skills, underscoring the critical need for targeted interventions focusing on healthy digital habits to safeguard cognitive-motor development in educational settings. The cross-sectional design precludes causal inference, and the high correlation between addiction severity and screen time indicates they are intertwined aspects of digital engagement.

## 1 Introduction

The pervasive integration of smartphones into adolescents’ daily lives has sparked a critical line of inquiry into the consequences of digital technology for cognitive development and mental health (Neophytou et al., 2021; Poujol et al., 2022). Adolescence represents a particularly sensitive developmental stage, marked by heightened neuroplasticity and the ongoing maturation of prefrontal cognitive control networks that support executive functions such as attention, planning, and self-regulation (Casey, 2015). At the same time, alarming evidence indicates that 70–80% of adolescents surpass recommended screen time thresholds, with average daily usage frequently exceeding 4 h (Oswald et al., 2020; Poujol et al., 2022). This surge in use has intensified concerns surrounding smartphone addiction, which is characterized by compulsive engagement, withdrawal symptoms, tolerance, and significant impairment in daily functioning (Kancharla et al., 2022; Prasetyadi and Gustaman, 2022). These patterns of use raise important questions about the potential for smartphone addiction to disrupt fundamental cognitive and motor processes crucial for effective learning and academic performance (Clemente-Suárez et al., 2024; Elhai et al., 2017).

The literature presents a complex yet concerning picture of the effects of smartphone addiction on specific cognitive domains, with the most consistent evidence emerging for attentional processes. A growing body of experimental and correlational research demonstrates that the constant influx of notifications and the habitual multitasking associated with addictive use fragment attention, resulting in measurable impairments in both selective and sustained attention (Ophir et al., 2009; Stothart et al., 2015). These deficits are considered to arise from cognitive overload induced by rapid task-switching, which progressively diminishes the ability to filter distractions and sustain focus on a single task over time. Neuroimaging studies further corroborate these behavioral findings, revealing altered functional connectivity within fronto-parietal networks critical for top-down attentional control among individuals exhibiting addictive smartphone use (Marciano et al., 2024).

In contrast, the relationship between smartphone addiction and higher-order abstract reasoning remains less well established and constitutes a notable area of inconsistency in the literature. Some studies report negative associations, indicating that excessive screen exposure may hinder the development of fluid intelligence and logical reasoning (Kancharla et al., 2022; Santos et al., 2022). Other investigations, however, yield null findings or even suggest context-dependent benefits, particularly when technology use is directed toward structured educational purposes (Hartanto et al., 2023; Toh et al., 2021). This heterogeneity implies that the effects on reasoning are not uniform but are likely moderated by factors such as the nature of digital content, the motivational context of use, and the extent to which screen time displaces other cognitively enriching activities (Hutton et al., 2024; Shanmugasundaram and Tamilarasu, 2023).

One of the most significant yet underexplored gaps lies in handwriting quality and speed, particularly in bilingual contexts such as Tunisia. Handwriting is not merely a motor activity but a complex process that integrates perceptual, cognitive, and executive functions, thereby supporting memory consolidation and deep learning (James and Engelhardt, 2012). Evidence indicates that excessive touchscreen use hampers graphomotor refinement (Sülzenbrück et al., 2011), with early childhood studies linking high screen exposure to poorer manual dexterity (Martzog and Suggate, 2022). This concern is especially pertinent in Tunisia, where adolescents must navigate a bilingual digraphic environment that involves switching between Arabic and Latin scripts, each with distinct orthographic demands (Amara et al., 2021; McNeil, 2024). Such bilingual orthographic complexity requires heightened spatial coordination, attentional control, and fine motor precision (Saiegh-Haddad and Henkin-Roitfarb, 2014). The cognitive load inherent in this bigraphism may be further undermined by attentional fragmentation and reduced fine-motor practice associated with smartphone addiction. As addictive smartphone use increasingly displaces traditional handwriting practice, the risk of pronounced declines in graphomotor speed and script quality—particularly across both Arabic and Latin writing systems—becomes increasingly acute in the Tunisian context (Lee, 2020; Osman, 2023).

Despite the growing body of research, progress in the field remains constrained by methodological heterogeneity and notable limitations, with a pronounced absence of studies in multilingual North African contexts. A central issue is the over-reliance on self-report measures and the frequent conflation of overall screen time with the distinct psycho-behavioral profile of addiction (Fabio et al., 2022; Yasin et al., 2023). Equally concerning is the complete lack of studies that integrate validated clinical scales for smartphone addiction with ecologically valid, paper-based assessments of cognition and graphomotor skills in bilingual populations. Such approaches are essential to avoid confounding effects of device-mediated testing and to capture the unique cognitive load associated with digraphia. In addition, environmental factors specific to Tunisia, including parental smartphone use patterns and pronounced urban–rural disparities in technology access and digital literacy, are likely to shape adolescents’ screen behaviors and addiction risk (Lai et al., 2022). These gaps highlight the need for research that accounts for both cultural and contextual influences while using more rigorous and ecologically grounded methodologies. While recent research has integrated objective screen time metrics with measures of problematic use in adult populations (Hartanto et al., 2023), the present study extends this approach to a critical developmental period, adolescence, within a unique bilingual North African context. This focus is particularly important given that adolescence represents a sensitive stage for prefrontal cognitive control network maturation (Casey, 2015), making this population potentially more vulnerable to digital technology effects. Furthermore, our study uniquely combines device-logged screen time with clinical addiction measures and ecologically valid paper-based assessments of both cognitive and graphomotor functions, addressing a significant gap in bilingual educational contexts where dual-script literacy imposes additional cognitive demands. This allows for a more comprehensive investigation of how digital behaviors relate to core neurocognitive and graphomotor functions essential for academic success.

Taken together, examining these variables simultaneously provides a more integrated perspective on adolescents’ digital behaviors and their potential cognitive consequences. By combining objective device-logged screen time, clinically validated measures of smartphone addiction, and ecologically valid paper-based assessments of attention and graphomotor performance, the present study enables the differentiation of the respective contributions of simple exposure to digital devices and the psycho-behavioral features of addiction. Furthermore, investigating these relationships within the Tunisian context, characterized by multilingual education and dual-script literacy (Arabic and Latin), offers novel insights into how digital behaviors interact with neurocognitive and motor processes in educational environments that have been largely overlooked in the existing literature.

To address these gaps, the present cross-sectional study examined the associations between smartphone addiction, conceptualized as a distinct clinical-behavioral construct, and key neurocognitive and motor functions in a well-defined cohort of Tunisian middle school adolescents aged 14 to 16 years within the educational context. By moving beyond generic measures of screen time, we directly compared adolescents meeting clinical criteria for smartphone addiction with their non-addicted peers across four critical domains: sustained attention, selective attention, handwriting speed and quality in Latin script, and abstract reasoning. In addition, we investigated whether smartphone addiction severity and total daily screen time independently predicted poorer performance in these domains, while statistically controlling for potential confounding variables such as age, sex, and body mass index. Based on prior evidence, we hypothesized that adolescents with smartphone addiction would demonstrate significant deficits across all measured domains and that a negative dose–response relationship would emerge for both addiction severity and screen exposure.

## 2 Methods

### 2.1 Sample

A sample of 270 adolescents (aged 14–16 years, M = 14.8, SD = 0.6$; 54% male students) was recruited via convenience sampling from three public middle schools in Tunisia during routine school health assessments between December 2023 and March 2024. The selection process began with an initial screening of 1,015 students using the Smartphone Addiction Scale-Short Version (SAS-SV) (Sfendla et al., 2018). To ensure a relevant study population, adolescents with no history of smartphone use were excluded at this stage.

To minimize confounding effects of the variables, rigorous exclusion criteria were implemented prior to enrollment. Individuals were excluded if they presented with: (a) clinically diagnosed psychiatric, neurological, or visual disorders; (b) current nicotine use; (c) chronic sleep disturbances (habitual sleep duration <7 or >9 h per night); (d) a body mass index (BMI) outside the 16.3–23.5 kg/m^2^ range, consistent with World Health Organization guidelines; or (e) incomplete data resulting from attrition. Following the application of these criteria, a final sample of 270 participants was confirmed. As detailed in Table 1, the resulting groups were comparable in baseline anthropometric and demographic characteristics, with no significant between-group differences in age, sex distribution, height, weight, or BMI (all *p* > 0.05).

**Table 1:** Participant characteristics.

Parameter	AS (*n* = 120)	NAS (*n* = 150)	Total (*n* = 270)	Min–Max	*U*	ES (*r*) [95% CI] *p*-value	
Age (years)	14.8 ± 0.6	14.8 ± 0.5	14.8 ± 0.5	14–16	8,767.5	0.02 [−0.05, 0.09]	0.714
Height (cm)	164.0 ± 7.5	163.6 ± 8.9	164.0 ± 8.3	148–185	8,403.5	0.06 [−0.03, 0.15]	0.349
Weight (kg)	54.0 ± 6.7	53.3 ± 7.1	53.6 ± 6.9	39–75	8,488.5	0.05 [−0.04, 0.14]	0.421
BMI (kg/m^2^)	19.90 ± 1.5	19.8 ± 1.3	19.8 ± 1.4	16.3–23.5	8,820.0	0.02 [−0.05, 0.09]	0.778

Groups did not differ significantly on demographic variables (all *p* > 0.05, Mann–Whitney *U*-test). Effect sizes (ESs) were interpreted as negligible (|*r*| < 0.1), small (0.1 ≤ |*r*| < 0.3), or moderate (0.3 ≤ |*r*| < 0.5). AS refers to adolescents with smartphone addiction, and NAS to non-addicted adolescents. BMI indicates body mass index; *U* is the Mann–Whitney *U* statistic; CI represents the confidence interval. All participants fell within the WHO-recommended BMI range for 14–16-year-olds (females: 18.03–23.15 kg/m^2^; males: 18.08–23.53 kg/m^2^).

### 2.2 Design

**Figure 1:**
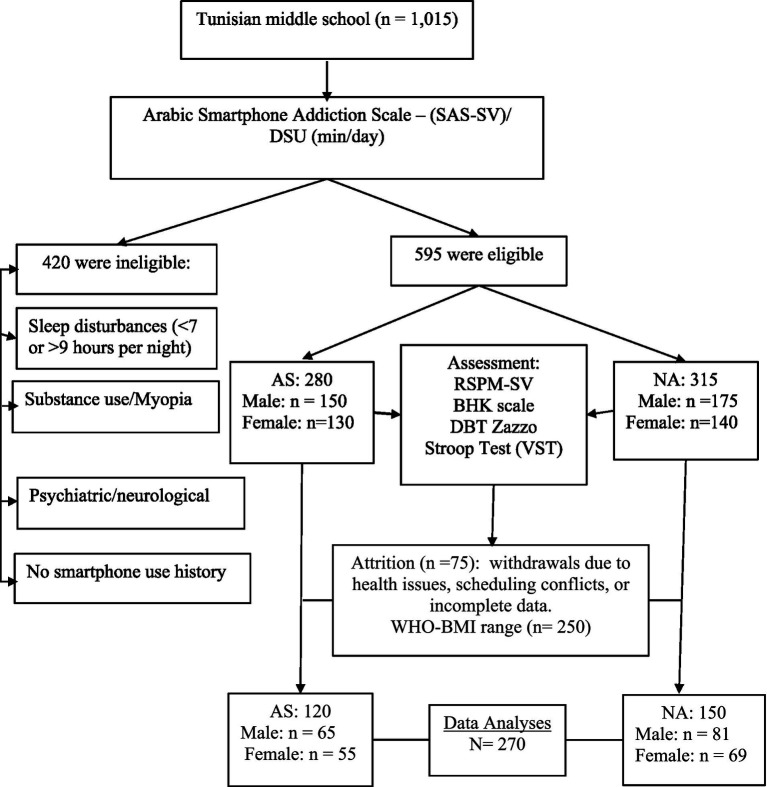
Flowchart experimental design and participant progression. SAS-SV refers to the Smartphone Addiction Scale-Short Version, while DSU denotes daily smartphone usage time. NAS represents non-addicted students, whereas AS stands for addicted students. The RSPM-SV (Raven Short-Version) assesses cognitive reasoning, and the BHK scale evaluates handwriting quality and speed as a measure of graphomotor skills. DBT; Zazzo corresponds to Zazzo’s Deux Barrages Test, which measures sustained attention. Finally, the Stroop Test (VST), or Victoria Stroop Test, is used to assess selective attention.

This study used a quasi-experimental, cross-sectional design to investigate the impact of smartphone usage patterns on adolescent executive-motor functions. Following the screening phase, participants were categorized into two distinct naturalistic cohorts: smartphone-addicted group (AS; *n* = 120) and non-addicted group (NAS; *n* = 150). This stratification was based on established gender-specific SAS-SV cutoff scores (≥31 for male students and ≥33 for female students). Figure 1 provides a comprehensive flowchart illustrating the experimental design and the progression of participants through each phase of the study, from initial screening to the final analytical groups.

### 2.3 Ethical considerations

The study was conducted in strict accordance with the ethical principles for medical research involving human subjects outlined in the Declaration of Helsinki. The research protocol received formal approval from the Regional Ethics Committee (Comité de Protection des Personnes SUD; Protocol No. 0554/2023) and complied with all relevant national regulations. Prior to participation, written informed consent was obtained from the parents or legal guardians of all adolescents, and verbal assent was secured from the participants themselves. All data were anonymized to ensure participant confidentiality, and individuals were informed of their right to withdraw from the study at any time without prejudice.

### 2.4 Procedures

Prior to executive-motor testing, smartphone usage data were collected. Following this data collection, participants completed four tasks in a fixed order on separate days: (1) a sustained attention task (Zazzo’s Deux Barrages Test, DBT), (2) a selective attention task (Victoria Stroop Test), (3) a handwriting task assessing graphomotor speed and quality (BHK handwriting scale), and (4) an abstract reasoning task (Raven’s Progressive Matrices).

Participants were selected using a convenience sampling approach from cooperating secondary schools. Ten trained instructors conducted all assessments in quiet classroom settings between 1:00 p.m. and 3:30 p.m., in accordance with research recommending that adolescent testing occur later in the day to align with peak circadian alertness and cognitive performance (Clarizio and Gill, 2022). Anthropometric data (height and weight) were recorded using a calibrated stadiometer. To minimize fatigue and order effects, each participant completed only one of the four tasks per day, and no more than 20 students were tested on any single day. Each test session lasted approximately 20 min.

Participants were instructed to abstain from caffeine and nicotine for at least 12 h before each testing session, with compliance verified through parental report, to avoid the acute effects of these substances on attention. Environmental conditions were kept constant in accordance with evidence-based recommendations for optimizing cognitive performance (Stephanidis et al., 2021). Two trained raters independently evaluated performance on these tasks, resulting in a high level of concordance in scoring. This is indicative of strong inter-rater reliability and procedural consistency.

### 2.5 Measures

#### 2.5.1 Smartphone addiction

Smartphone addiction was assessed using the Smartphone Addiction Scale-Short Version (SAS-SV; Kwon et al., 2013), a 10-item self-report tool rated on a 6-point Likert scale (1–6) covering daily life disturbances, withdrawal, cyberspace relationships, overuse, and tolerance. Scores range from 10 to 60, with gender-specific cutoffs (≥33 for females, ≥31 for male students, Sfendla et al., 2018). We used the validated Arabic version, administered via tablet (Google Forms) with researcher supervision to ensure standardized completion (Cronbach’s *α* = 0.89; test–retest *r* = 0.84).

#### 2.5.2 Daily smartphone use (DSU)

Daily smartphone use was obtained from device-based analytics (Apple iOS Screen Time or Android Digital Wellbeing), which log total daily screen time and app use. Data were recorded (minutes/day) over a 1-week period corresponding to cognitive testing to reduce self-report bias (Ellis et al., 2019). Previous research validated these built-in analytics as reliable objective indicators of smartphone behavior (Parry et al., 2021). Data from both iOS Screen Time and Android Digital Wellbeing were included. Participants using shared devices or with active scheduled restrictions (e.g., “Downtime” or “Focus” modes) during the recording period were excluded. Across the sample, the most frequent usage was observed in social media, messaging, and video-streaming applications.

#### 2.5.3 Abstract reasoning ability

Fluid intelligence was measured with a 15-item short form of Raven’s Standard Progressive Matrices (Langener et al., 2022; Raven et al., 1989), with 90 s/item time limits. Internal consistency was high (Cronbach’s *α* = 0.88).

#### 2.5.4 Graphomotor skills (handwriting)

Fine motor writing skills were assessed using the French BHK handwriting scale (Charles et al., 2004). Participants copied a standard text for 5 min. Two blinded raters scored handwriting quality across 13 criteria (ICC = 0.84, 95% CI [0.76–0.90]) and writing speed (letters/5 min; Godde et al., 2018).

#### 2.5.5 Selective attention

Selective attention and inhibition were evaluated with the Victoria Stroop Test (Spreen and Strauss, 1998), which includes baseline (dots), low-interference (color words), and high-interference (incongruent color–word) conditions. Completion time, errors, and an interference score were calculated.

#### 2.5.6 Sustained attention

Sustained attention was measured using Zazzo’s Deux Barrages Test (Zazzo, 1952), requiring cancellation of two target symbols over 10 min. Accuracy, speed, and overall performance indices (IN₂, V₂, and R₂) were computed.

### 2.6 Statistical analysis

Analyses were conducted using IBM SPSS Statistics Version 28. Normality of continuous variables was assessed using the Shapiro–Wilk test, and variables that violated normality (*p* < 0.05) were analyzed using non-parametric methods. Group differences were examined using Mann–Whitney *U*-tests, and associations were assessed with Spearman’s correlations (*ρ*). Effect sizes for group comparisons were reported as rank-biserial correlations (*r*), interpreted as small (0.10), medium (0.30), or large (0.50). A Bonferroni-adjusted *α* = 0.01 was applied to primary cognitive outcomes.

Sample size requirements were determined *a priori* using G*Power 3.1 (Faul et al., 2007). Given the study’s primary objective to examine the predictive value of smartphone addiction and screen time on cognitive-motor performance, a power analysis was conducted for a fixed-model multiple linear regression (*R*^2^ deviation from zero). Assuming a medium effect size (*f*^2^ = 0.15), a significance level of *p* < 0.05, and a desired statistical power (1 − *β*) of 0.80 with three predictors (age, sex, and BMI as covariates), the minimum required sample size was calculated to be 119 participants. Our final sample of 270 participants exceeds this threshold, providing sufficient power to detect meaningful associations and reducing the risk of type II errors.

Before conducting multiple linear regression analyses, all assumptions were evaluated. Normality of residuals was confirmed via Q-Q plots and the Shapiro–Wilk test (*p* > 0.05 for all models). Homoscedasticity was verified through visual inspection of residual plots, and linearity was assessed using partial regression plots. Multicollinearity was examined with variance inflation factors (VIFs), which indicated high collinearity between SAS-SV scores and daily screen time (VIF = 8.2), necessitating separate regression models. Independence of errors was confirmed using the Durbin-Watson test (1.89–2.11). Overall, all the assumptions were adequately satisfied.

The reliability of the assessment procedures was rigorously evaluated. Interrater reliability for the BHK handwriting scale was excellent, with an intraclass correlation coefficient (ICC) of 0.84 (95% CI: 0.76–0.90). Similarly, the scoring for the Victoria Stroop Test demonstrated a 97% interrater agreement. These results indicate a high degree of measurement consistency across independent raters.

## 3 Results

### 3.1 Participant characteristics

The sample comprised 270 adolescents, including a smartphone-addicted group (AS: *n* = 120) and a non-addicted group (NAS: *n* = 150). Mean age was approximately 14.8 years in both groups, with 54% male students and a similar socioeconomic status. No significant differences were found between the groups for age, height, weight, or BMI (all *p* > 0.05; Mann–Whitney *U*-test range: 8403.5–8820.0, *r* < 0.10) (Table 1). Exploratory analyses examining potential interaction effects (e.g., sex × addiction group, age × addiction group) on primary cognitive and motor outcomes revealed no significant interactions (all *p* > 0.05), indicating that the observed associations between smartphone addiction and cognitive-motor performance were consistent across demographic subgroups.

### 3.2 Group differences in cognitive and motor performance

Smartphone-addicted (AS) participants scored higher on the SAS-SV (M = 46.00 ± 8.00) than non-addicted (NAS) participants (*M* = 17.85 ± 9.74; *U* = 1.0, *p* < 0.001, *r* = 0.86) and had greater daily smartphone use (AS: 455.0 ± 105.0 min/day vs. NAS: 172.1 ± 108.6; *U* = 443.5, *p* < 0.001, *r* = 0.82) (Table 2).

**Table 2:** Motor and cognitive performance comparisons between AS and NAS.

Domain	Parameter	NAS (*n* = 150)	AS (*n* = 120)	Overall (*n* = 270)	Min–Max	*U*	*Z*	*p*-value	ES (*r*)
Smartphone use	SAS-SV score	17.85 ± 9.74	46.0 ± 8.0	30.47 ± 16.81	0–60	1.0	−14.133	**<0.001**	0.86
	DSU (min/day)	172.1 ± 108.6	455.0 ± 105.0	297.7 ± 176.6	0–615	443.5	−13.439	**<0.001**	0.82
Reasoning (RSPM S-V)	Correct items	8.01 ± 3.07	8.0 ± 3.0	7.99 ± 3.20	2–15	8,940.5	−0.094	0.925	0.01
	Time (s)	464.3 ± 141.9	471.0 ± 137.0	467.4 ± 139.6	200–765	8,701.0	−0.469	0.639	0.03
Sustained attention (DBT)	Speed (items/min)	19.7 ± 2.0	18.0 ± 2.0	18.9 ± 2.0	11.9–25.5	4,355.5	−7.288	**<0.001**	0.44
	Performance (correct items)	195.0 ± 19.4	177 ± 16	187.0 ± 20.0	118–248	3,970.5	−7.891	**<0.001**	0.48
	Inaccuracy (errors)	0.21 ± 0.08	0.0 ± 0.0	0.24 ± 0.08	0–0.52	4,355.5	−7.288	**<0.001**	0.44
Handwriting (BHK)	Quality (errors)	10.24 ± 7.09	13.0 ± 8.0	11.31 ± 7.72	0–38	7,462.5	−2.416	**0.016**	0.15
	Speed (letters)	394.3 ± 71.9	394 ± 54	394.2 ± 64.5	210–520	8,699.0	−0.472	0.637	0.03
Selective attention (VST)	Card D: RT (s)	19.14 ± 2.97	19.0 ± 3.0	19.15 ± 3.14	12.75–29.45	8,783.0	−0.340	0.734	0.02
	Card D: Errors	0.10 ± 0.30	0.0 ± 0.0	0.11 ± 0.31	0–1	8,850.0	−0.439	0.661	0.03
	Card W: RT (s)	29.70 ± 5.25	29.0 ± 5.0	29.59 ± 5.35	20.45–45.12	8,683.5	−0.496	0.620	0.03
	Card W: Errors	1.03 ± 1.21	1.00 ± 1.0	1.22 ± 1.24	0–5	7,087.5	−3.140	**0.002**	0.19
	Card C: RT (s)	49.19 ± 10.86	48.0 ± 10.0	48.60 ± 10.42	30.12–80.25	8,506.0	−0.775	0.438	0.05
	Card C: Errors	2.48 ± 1.94	3.0 ± 2.0	2.87 ± 2.05	0–8	6,790.5	−3.513	**<0.001**	0.21
	RT interference (s)	30.05 ± 9.07	29.0 ± 8.0	29.45 ± 8.51	14.23–61.70	8,384.0	−0.966	0.334	0.06
	Error interference (errors)	2.38 ± 1.80	3.0 ± 2.0	2.77 ± 1.91	0–8	6,656.5	−3.729	**<0.001**	0.23

Bolded *p*-values indicate statistical significance (*p* < 0.05). Effect sizes (ESs) are interpreted as small (*r* = 0.10), medium (*r* = 0.30), and large (*r* = 0.50). Abbreviations used include SAS-SV for Smartphone Addiction Scale–Short Version, DSU for daily smartphone usage, RSPM S-V for Raven’s Standard Progressive Matrices, DBT for Deux Barrages Test, BHK for French Handwriting Scale, VST for Victoria Stroop Test, and RT for reaction time.

**Figure 2:**
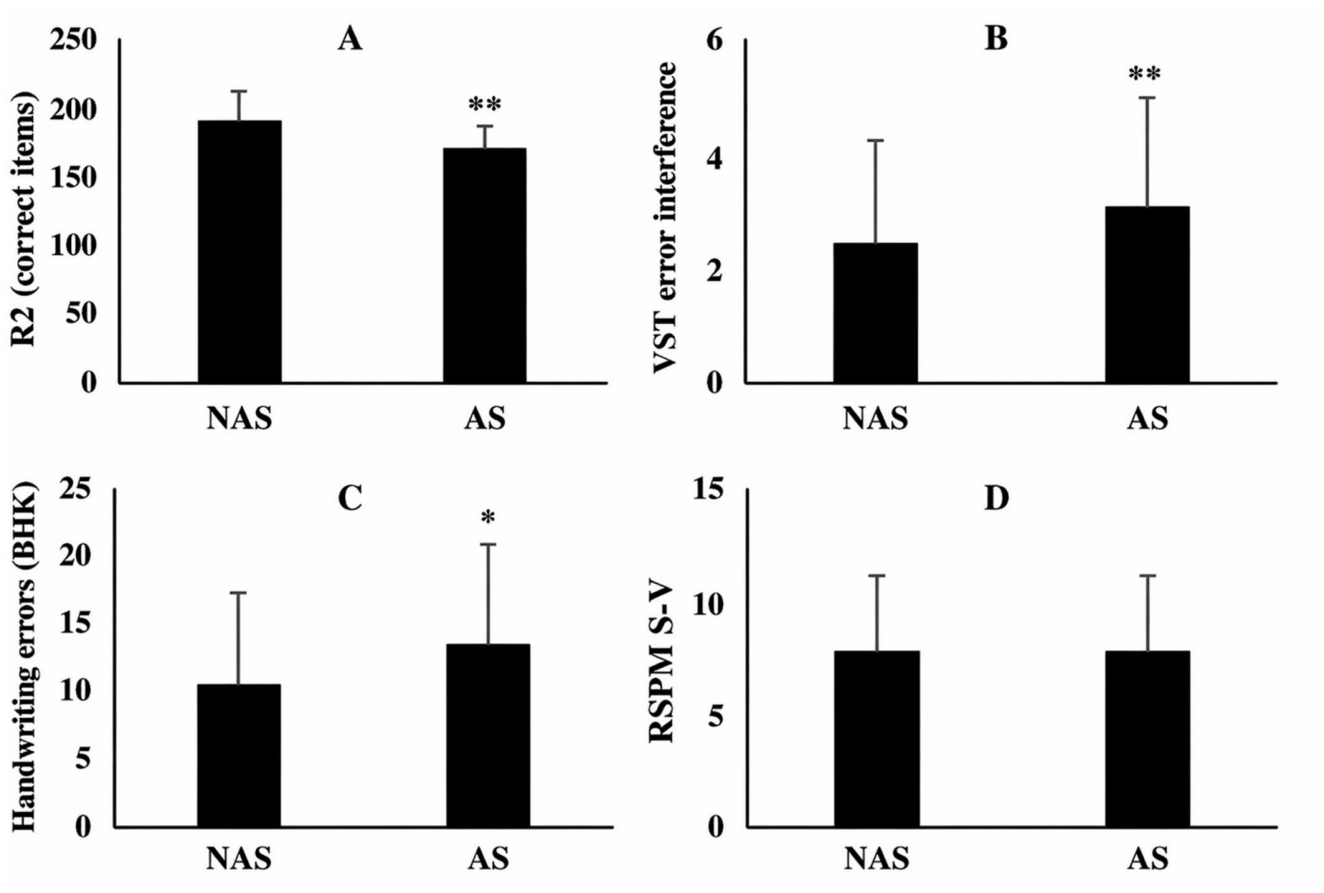
Group comparisons between NAS and AS across various cognitive scales [**(A)** DBT performance; **(B)** VST error interference; **(C)** BHK errors; and **(D)** RSPM-SV correct items]. *p < 0.05, **p < 0.001: significantly different compared with NAS. NA refers to non-addicted students, while AS stands for addicted students. RSPM-SV (Raven Short-Version) assesses cognitive reasoning, and the BHK scale evaluates handwriting quality and speed as a measure of graphomotor skills. DBT; Zazzo corresponds to Zazzo’s Deux Barrages Test, which measures sustained attention. The Stroop test (VST), or Victoria Stroop test, is used to assess selective attention. *R*^2^ represents performance in terms of the number of correct items.

No significant group differences were found in abstract reasoning, with similar scores on Raven’s Standard Progressive Matrices (NAS: 8.01 ± 3.07; AS: 8.00 ± 3.00; *U* = 8940.5, *p* = 0.925, *r* = 0.01) or completion times (NAS: 464.3 ± 141.9 s; AS: 471.0 ± 137.0 s; *U* = 8701.0, *p* = 0.639, *r* = 0.03) (Table 2 and Figure 2D).

Sustained attention performance differed between groups, with AS participants completing fewer items per minute (18.0 ± 2.0 vs. 19.7 ± 2.0; *U* = 4355.5, *p* < 0.001, *r* = 0.44), making fewer correct detections (177.0 ± 16.0 vs. 195.0 ± 19.4; *U* = 3970.5, *p* < 0.001, *r* = 0.48), and committing more inaccuracy errors (0.00 ± 0.00 vs. 0.21 ± 0.08; *U* = 4355.5, *p* < 0.001, *r* = 0.44) (Table 2 and Figure 2A).

Selective attention errors were higher in the AS group on the Victoria Stroop Test, especially under interference (Card C: 3.00 ± 2.00 vs. 2.48 ± 1.94 errors; *U* = 6790.5, *p* < 0.001, *r* = 0.21) and in the Stroop interference score (3.00 ± 2.00 vs. 2.38 ± 1.80; *U* = 6656.5, *p* < 0.001, *r* = 0.23). No group differences were observed in reaction times across Stroop conditions (all *p* > 0.05) (Table 2 and Figure 2B).

Handwriting quality was poorer in the AS group (13.0 ± 8.0 errors) compared to NAS (10.24 ± 7.09; *U* = 7462.5, *p* = 0.016, *r* = 0.15), while handwriting speed did not differ (394 ± 54 vs. 394.3 ± 71.9 letters; *U* = 8699.0, *p* = 0.637, *r* = 0.03) (Table 2 and Figure 2C).

### 3.3 Correlational analyses

Smartphone addiction severity and daily screen time were strongly correlated (Spearman *ρ* = 0.965, *p* < 0.001), indicating near-perfect collinearity (Table 3). Both indices showed similar associations with cognitive-motor performance. Higher smartphone use was associated with lower Raven’s reasoning scores (*ρ* = −0.286 for SAS-SV and −0.299 for screen time, *p* < 0.001), slower and less accurate sustained attention (*ρ* = −0.520 to −0.585, *p* < 0.001), more Stroop errors (*ρ* ≈ 0.17–0.40, *p* < 0.05), and more handwriting errors (*ρ* = 0.253–0.256, *p* < 0.001). Weak or non-significant associations were observed for handwriting speed (*ρ* ≈ −0.17) and reasoning task completion time (*ρ* ≈ −0.12, *p* ≈ 0.05).

**Table 3:** Spearman’s correlations (*ρ*) between smartphone use and motor-cognitive performance.

Domain	Measure	SAS-SV score	Daily smartphone usage
Smartphone use	SAS-SV score	1.000 (**<0.001**)	0.965 (**<0.001**)
	DSU (min/day)	0.965 (**<0.001**)	1.000 (**<0.001**)
Reasoning (RSPM S-V)	Score	−0.286 (**<0.001**)	−0.299 (**<0.001**)
	Time (s)	−0.119 (0.051)	−0.120 (0.050)
Sustained attention (DBT)	Speed (items/min)	−0.520 (**<0.001**)	−0.552 (**<0.001**)
	Performance (correct items)	−0.551 (**<0.001**)	−0.585 (**<0.001**)
	Inaccuracy (errors)	0.520 (**<0.001**)	0.552 (**<0.001**)
Selective attention (VST)	Card D: RT	0.180 (**0.003**)	0.205 (**0.001**)
	Card D: Errors	0.165 (**0.007**)	0.176 (**0.004**)
	Card W: RT	0.194 (**0.001**)	0.198 (**0.001**)
	Card W: Errors	0.382 (**<0.001**)	0.369 (**<0.001**)
	Card C: RT	0.174 (**0.004**)	0.183 (**0.003**)
	Card C: Errors	0.395 (**<0.001**)	0.390 (**<0.001**)
	RT interference	0.148 (**0.015**)	0.150 (**0.014**)
	Error interference	0.401 (**<0.001**)	0.396 (**<0.001**)
Handwriting (BHK)	Quality (errors)	0.253 (**<0.001**)	0.256 (**<0.001**)
	Speed (letters)	−0.158 (**0.010**)	−0.188 (**0.002**)

Bolded *p*-values indicate statistical significance (*p* < 0.05). Multicollinearity was observed between SAS-SV and daily screen time (*ρ* = 0.965, VIF = 8.2), so separate regression models were used to avoid inflated type I error. Effect size guidelines for Spearman’s *ρ* are 0.1 (small), 0.3 (medium), and 0.5 (large).

### 3.4 Regression models

Separate multiple linear regression models controlling for BMI, age, and sex were conducted for SAS-SV and daily screen time due to high collinearity (VIF = 8.2; Table 4). In these separate models, both predictors were significant predictors of sustained attention speed (*R*^2^ = 0.27–0.31, *β* = −0.52 to −0.55, *p* < 0.001), sustained attention performance accuracy (*R*^2^ = 0.30–0.34, *β* = −0.55 to −0.58, *p* < 0.001), and sustained attention errors (*R*^2^ = 0.27–0.31, *β* = 0.52 to 0.55, *p* < 0.001). Selective attention interference errors were also predicted in these models (*R*^2^ = 0.15–0.16, *β* ≈ 0.39–0.40, *p* < 0.001). Handwriting quality showed a smaller but significant association (*R*^2^ = 0.06–0.07, *β* ≈ 0.25–0.26, *p* < 0.001), while handwriting speed and abstract reasoning scores were minimally influenced (*R*^2^ = 0.02–0.04, *β* = −0.16 to −0.19, *p* ≤ 0.010).

**Table 4:** Separate linear regression models for SAS-SV and SMP-time predicting motor and cognitive performance.

Domain	Measure	Predictor	*β* [95% CI]	*p*-value	*R* ^2^	Adj. *R*^2^
Reasoning (RSPM S-V)	Score	SAS-SV	−0.28 [−0.35, −0.21]	**<0.001**	0.082	0.079
		DSU	−0.30 [−0.37, −0.23]	**<0.001**	0.089	0.086
Sustained attention (DBT)	Speed (items/min)	SAS-SV	−0.52 [−0.61, −0.43]	**<0.001**	0.270	0.268
		DSU	−0.55 [−0.64, −0.46]	**<0.001**	0.305	0.302
	Performance (correct items)	SAS-SV	−0.55 [−0.63, −0.47]	**<0.001**	0.304	0.301
		DSU	−0.58 [−0.66, −0.50]	**<0.001**	0.342	0.340
	Inaccuracy (errors)	SAS-SV	0.52 [0.43, 0.61]	**<0.001**	0.270	0.268
		DSU	0.55 [0.46, 0.64]	**<0.001**	0.305	0.302
Selective attention (VST)	Card C: Errors	SAS-SV	0.40 [0.31, 0.49]	**<0.001**	0.160	0.157
		DSU	0.39 [0.30, 0.48]	**<0.001**	0.152	0.149
	Error interference	SAS-SV	0.40 [0.31, 0.49]	**<0.001**	0.161	0.158
		DSU	0.40 [0.31, 0.49]	**<0.001**	0.157	0.154
Handwriting (BHK)	Quality (errors)	SAS-SV	0.25 [0.16, 0.34]	**<0.001**	0.064	0.061
		DSU	0.26 [0.17, 0.35]	**<0.001**	0.066	0.063
	Speed (letters)	SAS-SV	−0.16 [−0.25, −0.07]	**0.010**	0.025	0.022
		DSU	−0.19 [−0.28, −0.10]	**0.002**	0.035	0.032

Separate regression models were run for SAS-SV and daily smartphone use (DSU) due to high collinearity (VIF = 8.2). Cognitive performance metrics served as dependent variables, with either SAS-SV or DSU as the isolated predictor. The standardized regression coefficient (*β*) indicates both the strength (effect size) and direction of the association. *R*^2^ represents the proportion of variance explained by the predictor, interpreted as small (0.01–0.09), medium (0.09–0.25), or large (>0.25). Adjusted *R*^2^ accounts for sample size and number of predictors. Bolded *p*-values denote statistical significance (*p* < 0.05).

## 4 Discussion

This study examined associations between smartphone addiction and cognitive–motor performance in Tunisian adolescents. The findings indicate that smartphone addiction is associated with poorer sustained and selective attention and reduced handwriting quality, while handwriting speed and abstract reasoning remain largely unaffected.

Regression analyses showed that addiction severity and daily screen time strongly predicted sustained attention deficits (27–34% variance explained) and were moderately associated with selective attention errors (15–16%) and handwriting quality (6–7%), with minimal effects on handwriting speed and abstract reasoning (2–4%). The high correlation between these two digital engagement measures (*ρ* = 0.965) indicates they represent closely related aspects of modern smartphone use, both capturing critical dimensions of potentially detrimental digital engagement. These results suggest that excessive smartphone use selectively impairs attentional and graphomotor functions while sparing other cognitive domains.

The decline in sustained attention observed aligns with neurocognitive research linking excessive digital media use to diminished activation in the anterior cingulate cortex, a key region for attentional control and error monitoring (Lin et al., 2022). Behavioral evidence supports that frequent media switching impairs attentional stability, with smartphone addiction severity explaining a substantial proportion of variance in sustained attention deficits (Nagata et al., 2024). These findings underscore the vulnerability of sustained attentional processes to prolonged digital engagement.

Selective attention impairments, reflected in increased errors under interference conditions despite preserved reaction times, suggest a disruption in top-down inhibitory control mechanisms. This dissociation aligns with previous studies where heavy digital media users maintain processing speed but exhibit reduced accuracy, potentially due to a habituation to rapid stimuli that undermines error monitoring (Wilmer et al., 2017). These findings align with neurofunctional observations linking digital addiction to striatal hyperactivity and prefrontal hypoactivation, a pattern indicating a bias toward automated responses at the expense of cognitive regulation (London, 2020). At a neurochemical level, these functional changes may be driven by alterations in the brain’s reward systems. Excessive smartphone use in adolescents has been linked to altered dopaminergic and opioid functioning, reflecting dysregulated reward processing (Leal et al., 2025; Li, 2023). Dysfunctional reward signaling, characterized by hyperanticipation of digital rewards and blunted feedback from real-world activities, can exacerbate stress-related overuse and contribute to the observed deficits in inhibitory control (Qi et al., 2025).

Supporting this finding, electroencephalography studies confirmed reduced prefrontal engagement during tasks requiring response regulation, suggesting repetitive practice of rapid gestures (tapping and swiping) reinforces automaticity over voluntary control (Mac-Auliffe et al., 2021). Within the attentional control theory framework, these smartphone-related distractions and neural changes likely deplete cognitive resources, impairing task-focused behavior (Eysenck et al., 2007).

The interference effects observed support the hypothesis that multitasking with digital devices overloads working memory, diminishing inhibitory control (Ophir et al., 2009). The neurobiological underpinnings of this may involve increased connectivity within salience and habit networks (e.g., the putamen), which have been shown to mediate compulsive use and mood-related symptoms (Lee et al., 2024). In the Tunisian bilingual educational context, where adolescents manage dual-script literacy and demanding curricula, cognitive load may be amplified, increasing susceptibility to attentional disruptions.

Research shows that bilingual individuals who manage dual scripts may experience elevated cognitive demands that influence executive control, with biscriptal bilinguals demonstrating greater attention efficiency than monoscriptal peers (Yang et al., 2019). The reduction in handwriting quality, but not speed, suggests that, while automated motor execution remains intact, fine motor control and attentional modulation of handwriting suffer in smartphone-addicted adolescents.

Contextual factors likely modulate these associations. Tunisia’s high adolescent smartphone penetration combined with bilingual education imposes unique cognitive demands, particularly in handwriting tasks requiring dual-script proficiency. Prior research shows that bilingual digraphia increases cognitive load during writing, and digital multitasking may be further compromised (Saiegh-Haddad and Henkin-Roitfarb, 2014). Additionally, environmental influences such as parental smartphone behavior and urban–rural disparities in technology access shape adolescents’ screen use patterns and addiction risk (Lai et al., 2022). Critically, longitudinal data confirm that high addictive-use trajectories predict poorer mental health outcomes (Xiao et al., 2025), underscoring the public health importance of the cognitive-motor vulnerabilities identified in our study.

This dissociation aligns with motor interference models positing that touchscreen overuse hampers graphomotor refinement (Sülzenbrück et al., 2011). Early childhood studies linking high screen exposure to poorer manual dexterity further support this relationship (Martzog and Suggate, 2022). Moreover, bilingual orthographic complexity requires enhanced spatial and attentional coordination, potentially exacerbating handwriting difficulties in this population (Saiegh-Haddad and Henkin-Roitfarb, 2014).

The absence of significant group differences in abstract reasoning suggests that fluid intelligence may be resilient to short-term environmental influences of smartphone use (Lilienfeld et al., 2014). However, the modest negative correlations with addiction scores hint at cumulative cognitive effects over time. Longitudinal data indicate that prolonged media exposure can reduce problem-solving efficiency (Luo et al., 2024). Compensatory factors, such as engagement with cognitively demanding digital games, may also attenuate deficits in reasoning by stimulating executive functions and frontal lobe activity (Alho et al., 2022).

These findings corroborate cognitive load theory, which posits that managing multiple information streams overwhelms working memory capacity, impairing performance (Sweller, 1988). Baddeley’s working memory model similarly explains how constant digital engagement saturates central executive resources necessary for sustained attention, inhibitory control, and fine motor coordination (Baddeley and Hitch, 2000).

It is crucial to consider the interpretative limitations of our cross-sectional findings. The observed associations do not imply causation, and reverse or bidirectional pathways are plausible. For instance, adolescents with pre-existing vulnerabilities in attentional control or executive functioning may be more prone to developing problematic smartphone use patterns as a means of stimulation or regulation (Kasturiratna and Hartanto, 2025). Future longitudinal research is essential to disentangle the temporal precedence and potential bidirectional relationships between digital engagement and cognitive-motor functions.

While this study provides valuable insights into the intersection of smartphone use and adolescent motor-cognitive function, several methodological limitations warrant consideration. First, the cross-sectional design precludes causal inference. Second, although device-logged screen-time metrics are validated as reliable indicators of smartphone behavior, they may underestimate use during offline periods or on secondary devices not running the native operating system (Parry et al., 2021). Nevertheless, these metrics are a methodological improvement over self-reported screen time, which is prone to substantial error (Ellis et al., 2019). Third, while objective, these metrics do not capture the qualitative nature of smartphone use; recreational apps predominated (e.g., social media and video streaming), but different content types may have distinct effects. Fourth, the SAS-SV, though validated in Arabic, lacks cutoffs specifically normed for Tunisian adolescents; we applied widely used Moroccan cutoffs (Sfendla et al., 2018), which may not fully reflect local cultural patterns. Fifth, multiple statistical comparisons increase the risk of Type I error, although a conservative Bonferroni-adjusted *α* = 0.01 was applied for primary outcomes. Sixth, smartphone addiction was assessed via self-report rather than through a clinical interview, which represents a methodological limitation. Finally, the high correlation between addiction scores and screen time, while statistically addressed, indicates conceptual overlap, complicating the disentanglement of their effects. Longitudinal studies are needed to clarify the long-term cognitive-motor consequences of smartphone addiction in adolescents.

## 5 Conclusion

In this cross-sectional study involving adolescents aged 14–16 years, smartphone addiction was significantly associated with poorer performance in specific cognitive-motor domains. Analyses revealed that higher addiction scores and greater daily screen time were correlated with deficits in sustained and selective attention, as well as reduced handwriting quality. A clear dose–response gradient was observed, wherein increasing levels of smartphone use and addiction severity were linked to progressively worse outcomes in these domains. In contrast, no such associations were found with abstract reasoning or handwriting speed. It must be noted that the cross-sectional design prevents causal inference, and the relationships observed may be bidirectional in nature.

These findings highlight associations between smartphone addiction and specific cognitive-motor vulnerabilities during a critical developmental period. The strength of the associations and the presence of a dose–response pattern suggest a potentially important public health concern. This is particularly relevant in bilingual educational settings, which demand high levels of attentional control and graphomotor precision. Developing pedagogical frameworks that preserve core cognitive abilities while fostering adaptability and resilience is crucial as digital immersion becomes increasingly pervasive.

## Data Availability

The raw data supporting the conclusions of this article will be made available by the authors, without undue reservation.
